# Neuralgia Caused by Granules of Osteo Dentine in Pulp Cavities of Teeth

**Published:** 1874-01

**Authors:** A. Miller


### NEURALGIA CAUSED BY GRANULES OF OSTEO DENTINE IN PULP CAVITIES OF TEETH.

BY A. MILLER.

This is a very obscure disease and difficult to diagnose, owing to the absence of external signs, which might at all times indicate the presence of granules in the pulp. The following four cases have come under my treatment since last September, and thinking that a short and even imperfect history may be interesting to many readers of the REGISTER, I have been induced to send it for publication. The first case, that of a lady, Mrs. R., aet. 30, who had suffered more or less at intervals for about a year, was treated by a physician for idiopathic neuralgia, without any benefit. He being unable to discover any cause. Pain was confined to left side of face and head, with occasional darting pain in the neck, shoulder and arm. Examined all the teeth on the left side carefully, also on right side. Many of the teeth contained small cavities, nerves not exposed. Tapped each tooth with an instrument, and patient experienced, as she said, a "curious" feeling in left superior second molar. With small drill entered through cavity of decay into pulp chamber; patient experienced but little pain. Enlarged opening, and with fine-pointed instrument made examination to discover the little bundle of semi-bone-like material in pulp cavity. Removed it, and the patient has not had a symptom of pain since, now over two months ago.

The second case, that of a man, Mr. S., aet 27; had pain for long time in side of face and head, and at times patient stated all the teeth on the right side seemed to ache; wanted some of his teeth pulled right out; supposed that some one of the teeth on right was the cause of the trouble; most all were filled with gold or amalgam, and were sensitive to jet of cold water thrown from a syringe, except second superior molar on right side which contained an amalgam filling. Removed filling and found floor of cavity solid and not in the least sensitive; drilled into pulp cavity, and applied creosote. Patient said he was relieved; returned next day, had suffered all the previous night; pain worse than ever, recurring at shorter intervals during day. Proceded to open the tooth at once, and found "granules" occupying almost entire pulp cavity. This was a beautiful specimen; six or eight little, almost transparent balls, all compacted and apparently as hard as glass. The patient experienced some pain during the removal, but has had no return of the disease since.

The third case, that of a lady who, as she told me, had pain in her jaw and face for several weeks. Her medical adviser failed to give relief; I at the first sitting decided which tooth caused the trouble and diagnosed granules in pulp cavity; there was small cavity in the tooth, and very sensitive nerve not exposed; applied arsenic and dismissed patient. Next day removed the largest specimen of granules that I have ever seen. They appeared united, but by rolling between thumb and finger the little nodules separated, and under a magnifying glass were as round as shot; perfectly smooth and of a clear amber color. The lady has been free from neuralgia since that time.

The last case, a man came to me complaining about as the other patients, could not localize the pain, was much worse at night, about three attacks during the day; came to my office four times before I decided which tooth, if any, caused the mischief, when I finally removed the amalgam filling from second left superior molar ; endeavored to drill into pulp cavity, but after a few turns of drill found the tooth so exceedingly sensitive that I applied paste, which I let remain 24 hours, when I had no trouble in opening pulp chamber and extracted therefrom a small bundle of granules. There was very little nerve or pulp except in palatal root, no pain since. I have not been as explicit in details as I might, for fear of making too lengthy an article ; but if there is any point on which any one would like further information pertaining to the above cases, I shall be pleased to inform them the best I can.

